# A powerful nonparametric method for detecting differentially co-expressed genes: distance correlation screening and edge-count test

**DOI:** 10.1186/s12918-018-0582-x

**Published:** 2018-05-16

**Authors:** Qingyang Zhang

**Affiliations:** Department of Mathematical Sciences, University of Arkansas, Fayetteville, AR 72701 USA

**Keywords:** Distance correlation, Edge-count test, Differential co-expression, Breast cancer subtypes, Pathway analysis, The cancer genome atlas

## Abstract

**Background:**

Differential co-expression analysis, as a complement of differential expression analysis, offers significant insights into the changes in molecular mechanism of different phenotypes. A prevailing approach to detecting differentially co-expressed genes is to compare Pearson’s correlation coefficients in two phenotypes. However, due to the limitations of Pearson’s correlation measure, this approach lacks the power to detect nonlinear changes in gene co-expression which is common in gene regulatory networks.

**Results:**

In this work, a new nonparametric procedure is proposed to search differentially co-expressed gene pairs in different phenotypes from large-scale data. Our computational pipeline consisted of two main steps, a screening step and a testing step. The screening step is to reduce the search space by filtering out all the independent gene pairs using distance correlation measure. In the testing step, we compare the gene co-expression patterns in different phenotypes by a recently developed edge-count test. Both steps are distribution-free and targeting nonlinear relations. We illustrate the promise of the new approach by analyzing the Cancer Genome Atlas data and the METABRIC data for breast cancer subtypes.

**Conclusions:**

Compared with some existing methods, the new method is more powerful in detecting nonlinear type of differential co-expressions. The distance correlation screening can greatly improve computational efficiency, facilitating its application to large data sets.

## Background

The vast majority of human diseases are complex diseases, in the sense that they are not the consequence of an abnormality of a single gene, but a result of changes in many genes. Thanks to the rapid advance of high-throughput technologies, researchers nowadays can investigate the association between a disease and tens of thousands of genes simultaneously. Two types of analysis, namely differential expression (DE) analysis and differential co-expression (DCE) analysis, have been extensively applied in genetic association studies [1–4]. Differential expression analysis targets genes with differential expression levels in different phenotypes, while DCE analysis detects gene pairs or gene sets that are differentially associated or regulated in different groups. Over the past years, there have been considerable tools developed for DE analysis and other similar analyses such as differential methylation (DM) analysis. One can refer to Soneson and Delorenzi (2013) [5] for a comprehensive review and comparison of several most popular tools including *edgeR*, *DESeq*, *TSPM*, *baySeq*, *EBSeq* and *ShrinkSeq*. Despite the success of DE analysis, the progress on DCE analysis is relatively slow partially due to the combinatorial nature of the problem and the lack of powerful statistical test for comparing multi-dimensional patterns.

Current DCE analyses are mostly relied on Pearson’s correlation coefficient [1, 2, 6, 7], which is sensitive to outliers and only measures the strength of linear dependence. Some modified measures such as Spearman’s correlation and biweight midcorrelation [1] are more robust to outliers, but still unable to capture nonlinear changes in co-expression. In this paper, we introduce a new method to generally test for DCE gene pairs without assuming linear or monotonic relation between genes. First of all, it is important to emphasize that the objective of this work is to search for differential co-expressions of single gene pairs, which is different from objective of approaches that set out to find modules of differentially co-expressed genes. To begin with, we give the formal definitions of gene co-expression and DCE genes: *“The co-expression of two genes is defined as the dependence between their expression levels. If the dependency structure in one phenotype is different from that in another, the two genes are called DCE genes”* [8]. For computational simplicity, most existing methods assume that genes are jointly normally distributed, i.e., the correlations between genes are linear. Under this assumption, the DCE testing is equivalent to testing the equality of two correlation coefficients, which can be formulated as the following hypothesis testing

$$H_{0}: \rho_{1}=\rho_{2} \quad \text{vs} \quad H_{\alpha}: \rho_{1}\neq\rho_{2},$$

where *ρ*_1_ and *ρ*_2_ represent the true correlation coefficients between gene A and gene B in two phenotypes. Let *r*_1_ and *r*_2_ be the sample correlation coefficients, by Fisher’s z-transformation, we have

$$z_{1}:=\frac{1}{2}\log\frac{1+r_{1}}{1-r_{1}}\rightarrow N\left(\frac{1}{2}\log\frac{1+\rho_{1}}{1-\rho_{1}}, \frac{1}{\sqrt{n_{1}-3}}\right), $$

$$z_{2}:=\frac{1}{2}\log\frac{1+r_{2}}{1-r_{2}}\rightarrow N\left(\frac{1}{2}\log\frac{1+\rho_{2}}{1-\rho_{2}}, \frac{1}{\sqrt{n_{2}-3}}\right), $$

where *n*_1_ and *n*_2_ stand for the sample sizes of two phenotypes. A routine two-sample z-test can then be directly applied to evaluate the significance:

$$p\text{-value}=2\mathrm{P}\left(Z>\frac{\left|z_{1}-z_{2}\right|}{\sqrt{\frac{1}{n_{1}-3}+\frac{1}{n_{2}-3}}}\right), $$

where *Z* represents a standard normal random variable.

The method described above is simple as the calculation only involves product-moment correlations, and it generally works well for linearly dependent genes. However, the assumption of joint normality is not realistic as the gene expression data could strongly deviate from normality. To this end, we relax the normal assumption and reformulate the DCE search as a general statistical comparison between two joint distributions, so that the DCE genes, based on their definition, can be tested through the following hypothesis setup:

$$H_{0}: \mathbf{F}^{*}_{1}=\mathbf{F}^{*}_{2} \quad \text{vs}\quad H_{\alpha}: \mathbf{F}^{*}_{1}\neq\mathbf{F}^{*}_{2}, $$

where $\mathbf {F}^{*}_{1}$ and $\mathbf {F}^{*}_{2}$ represent the joint distributions of genes A and B in two phenotypes after the quantile normalization. By quantile normalization, the marginal distributions match across groups, so that one can test for the difference between two dependency structures (in spirit, it is same as comparing two copula densities, $\frac {f_{1}(x, y)}{\int f_{1}(x, y)dx \int f_{1}(x, y)dy}$ and $\frac {f_{2}(x, y)}{\int f_{2}(x, y)dx \int f_{2}(x, y)dy}$). A significant discrepancy between $\mathbf {F}^{*}_{1}$ and $\mathbf {F}^{*}_{2}$ indicates differential co-expression in two phenotypes.

It should be noted that the test proposed here does not rely on any parametric assumption but generally targets all types of DCE. One can explicitly test *H*_0_ with a recently developed edge-count test [9]. However, unlike the Pearson’s correlation method, the new test requires several intermediate steps including the calculation of minimum spanning trees, therefore it could be less efficient when applied to large-scale data. To overcome this difficulty, we use the distance correlation measure to screen out non-coexpressed (independent) gene pairs before the edge-count test, so that the search space can be greatly reduced. The distance correlation measure has appealing theoretical properties and can generally capture nonlinear associations. On the whole, we put forward a complete framework for DCE analysis which is effective and applicable to large-scale expression data.

The rest of the paper is structured as follows: Section “Methods” reviews the technical details of distance correlation screening and edge-count test. Simulation studies are performed to compare the edge-count test with two existing approaches based on Pearson’s correlation and mutual information. In Section “Results”, we apply this new approach to the Cancer Genome Atlas (TCGA) data as well as the METABRIC data for the DCE analysis between four subtypes of breast cancer. We discuss the strengths and some possible extensions of the new approach in Section“Discussion” and conclude this paper in Section“Conclusions”.

## Methods

### Distance correlation screening

Our screening step is based on distance correlation (DC), which is a measure of dependence between two random vectors, not necessarily of same dimension [10]. For given random vectors **X** and **Y**, if we let *ϕ*_*x*_(*t*) and *ϕ*_*y*_(*s*) be the respective characteristic functions, then the distance covariance between *X* and *Y* can be defined as follows:

1 $$ {}\text{dCov}^{2}(\pmb{X}, \pmb{Y}) =\!\! \int_{R^{d_{x}+d_{y}}}\!\frac{||\phi_{\pmb{x}, \pmb{y}}(\pmb{t}, \pmb{s}) \,-\, \phi_{\pmb{x}}(\pmb{t})\phi_{\pmb{y}}(\pmb{s})||^{2}\omega(\pmb{t}, \pmb{s})\!}{c_{d_{x}}c_{d_{y}}||\pmb{t}||^{1+d_{x}}_{d_{y}}||\pmb{s}||^{1+d_{y}}_{d_{y}}}d\pmb{t}d\pmb{s},  $$

where *d*_*x*_ and *d*_*y*_ are the dimensions of *X* and *Y*, $c_{d_{x}}=\frac {\pi ^{(1+d_{x})/2}}{\Gamma \{(1+d_{x})/2\}}$ and $c_{d_{y}}=\frac {\pi ^{(1+d_{y})/2}}{\Gamma \{(1+d_{y})/2\}}$. Unless otherwise specified, $||\pmb {z}||_{d_{z}}$ denotes the Euclidean norm of $\pmb {z}\in \mathbb {R}^{d_{z}}$, and $||\phi ||^{2} = \phi \bar {\phi }$ for a complex-valued function *ϕ* and its conjugate $\bar {\phi }$.

Similar as Pearson’s correlation coefficient, the DC between *X* and *Y* is defined as a rescaled distance covariance:

2 $$ \text{dCor}{(\mathbf{X}, \mathbf{Y})} = \frac{\text{dCov}(\pmb{X}, \pmb{Y})}{\sqrt{\text{dCov}(\pmb{X}, \pmb{X})\text{dCov}(\pmb{Y}, \pmb{Y})}}.  $$

Generally, we have 0≤dCor(**X**,**Y**)≤1, which is different from Pearson’s correlation. One remarkable property of DC is that dCor(**X**,**Y**)=0 if and only if *X* and *Y* are independent [11–13], indicating that DC can also measure nonlinear associations. With random samples {(*X*_*i*_,*Y*_*i*_),*i*=1,…,*n*}, a natural estimator of dCov(*X*,*Y*) can be obtained as follows:

3 $$ \widehat{\text{dCov}}^{2}(\pmb{X},\pmb{Y}) = \frac{1}{n^{2}}\sum\limits_{i=1}^{n}\sum\limits_{j=1}^{n}A_{ij}B_{ij},  $$

where

$$A_{ij}=a_{ij}-\bar{a}_{i}-\bar{a}_{j}+\bar{a},$$

$$B_{ij}=b_{ij}-\bar{b}_{i}-\bar{b}_{j}+\bar{b},$$

if we let $\phantom {\dot {i}\!}a_{ij}=||\pmb {X}_{i}-\pmb {X}_{j}||_{d_{X}}$, $\bar {a}_{i}=\frac {1}{n}\sum \limits _{k=1}^{n}||\pmb {X}_{k}-\pmb {X}_{i}||_{d_{X}}$, $\bar {a}_{j}=\frac {1}{n}\sum \limits _{l=1}^{n}||\pmb {X}_{l}-\pmb {X}_{j}||_{d_{X}}$, $\bar {a}=\frac {1}{n^{2}}\sum \limits _{k=1}^{n}\sum \limits _{l=1}^{n}||\pmb {X}_{l}-\pmb {X}_{k}||_{d_{X}}$, $b_{ij}=||\pmb {Y}_{i}-\pmb {Y}_{j}||_{d_{Y}}\phantom {\dot {i}\!}$, $\bar {b}_{i}=\frac {1}{n}\sum \limits _{k=1}^{n}||\pmb {Y}_{k}-\pmb {Y}_{i}||_{d_{Y}}$, $\bar {b}_{j}=\frac {1}{n}\sum \limits _{l=1}^{n}||\pmb {Y}_{l}-\pmb {Y}_{j}||_{d_{Y}}$, $\bar {b}=\frac {1}{n^{2}}\sum \limits _{k=1}^{n}\sum \limits _{l=1}^{n}||\pmb {Y}_{l}-\pmb {Y}_{k}||_{d_{Y}}$. The sample estimate of DC can be obtained immediately:

4 $$ \widehat{\text{dCor}}(\pmb{X},\pmb{Y}) = \frac{\widehat{\text{dCov}}(\pmb{X},\pmb{Y})}{\sqrt{\widehat{\text{dCov}}(\pmb{X},\pmb{X})\widehat{\text{dCov}}(\pmb{Y},\pmb{Y})}}.  $$

One can test for significance of DC using an approximate t-test proposed by Szekely and Rizzo (2013) [13], which was implemented in R package *energy* [14]. Szekely and Rizzo (2013) established the following result under high dimensions

$$\mathcal{T}_{n}=\sqrt{v-1}\frac{\mathcal{R}^{*}_{n}(\pmb{X},\pmb{Y})}{\sqrt{1-(\mathcal{R}^{*}_{n}(\pmb{X},\pmb{Y}))^{2}}}\rightarrow t_{df=v-1},$$

where $\mathcal {R}^{*}_{n}(\pmb {X},\pmb {Y})$ represents a modified distance correlation between *X* and *Y* (see Szekely and Rizzo (2013), Eq 2.10, p.197) and $v=\frac {n(n-3)}{2}$. Here, it is worth noting that although the t-approximation above is derived under high dimensions, it also works well for low-dimension cases (in our problem, dimensions of *X* and *Y* both equal one for each test). To evaluate the performance of the t-approximation under dimension one, we consider two independence settings

- Setting 1: *X*_*i*_∼N(0,1), *Y*_*i*_∼N(0,2), *i*=1,2,…,50,
- Setting 2: *X*_*i*_∼Uniform(0,1), *Y*_*i*_∼Uniform(0,2), *i*=1,2,…,50.

**Fig. 1:**
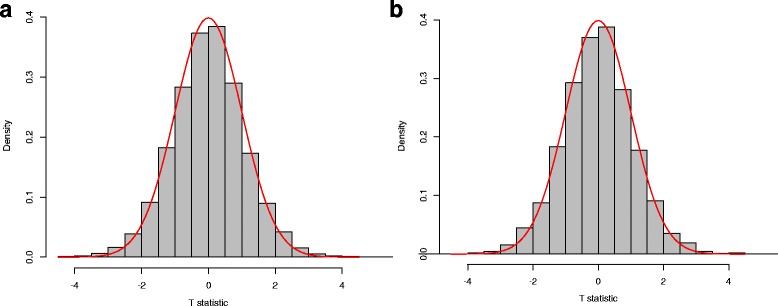
Finite sample performance of the t approximation under low dimensions (*d*_*x*_=*d*_*y*_=1): (**a**) normal setting and (**b**) uniform setting. The histograms represent the sample distributions of the test statistic $\mathcal {T}_{n}$ under independence null, and the curves are corresponding t curves

**Fig. 2:**
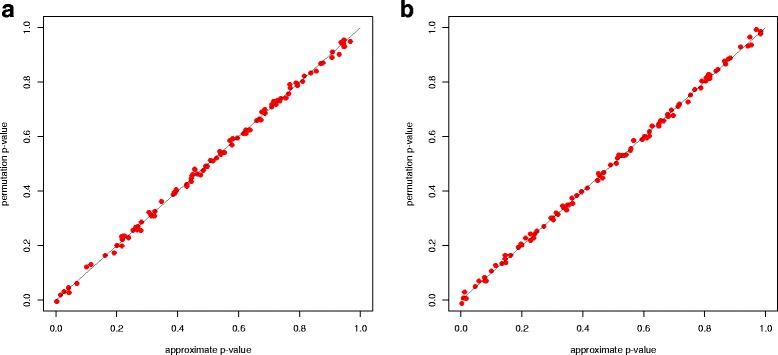
Accuracy of the *p*-value approximation by asymptotic t distribution under: (**a**) normal setting and (**b**) uniform setting. The x-axis is the approximate *p*-value and y-axis is the permutation *p*-value based on 10,000 random shuffles

For each setting, we generated 10,000 data sets and calculated the test statistic $\mathcal {T}_{n}$ for each data set. Figure 1 compared the sample distribution of $\mathcal {T}_{n}$ with the asymptotic t distribution (close to a standard normal distribution as the degree of freedom *v*−1 is generally large). Futhermore, we compared the approximate *p*-value with the permutation *p*-value (based on 10,000 random shuffles) in 100 replications. As shown in Fig. 2, the approximate *p*-values are very close to the permutation *p*-values, indicating a satisfactory performance of the t-approximation under low dimensions.

The distance correlation measure has been applied in previous genomic studies to quantify gene co-expressions [15]. Besides DC, there are several measures that can pick up nonlinear dependence between variables, although each of them has its own practical limitations. Clark (2013) [16] empirically compared six popular measures including Pearson’s correlation, Spearman’s correlation, distance correlation, mutual information (MI), maximum information coefficient (MIC) and Hoeffding’s D under a variety of different settings, and it was found that the six methods perform almost equally well in detecting the linear correlation. However, under the nonlinear dependence, the distance correlation and MIC performed notably better than the other measures. There are two considerations that lead to the choice of DC instead of MIC in our analysis. First, DC is straightforward to calculate and not an approximation while MIC relies on a user-defined number of grids for approximation. Second, as pointed out in some recent studies [17, 18], the DC exhibits more statistical power than MIC under moderate or small sample sizes.

### Edge-count test

Our testing step is to compare two multivariate distributions (dimension is 2 in DCE analysis). In statistics literature, there are mainly two types of multivariate tests, namely the multi-dimensional Kolmogorov-Smirnov (KS) test [19] and edge-count test [20, 21]. These two methods, however, both have practical limitations when applied to real data. For instance, KS test is very conservative, i.e., the null hypothesis is too often not rejected. Also, by the brute force algorithm, the application of multi-dimensional KS test can be prohibitively computationally intensive. The edge-count test is easy to implement but it is known to be problematic under the location and scale alternatives. Recently, Chen and Friedman [9] developed a modified version of edge-count test, which works properly under different alternatives and exhibits substantial power gains over existing edge-count tests. Similar as other edge-count tests, the new test is based upon a similarity graph such as minimum spanning tree (MST, [22]) that is constructed over the pooled samples from different groups. Generally, if two groups have different distributions, samples would be preferentially closer to others from the same group than those from the other group, therefore the edges in the MST would be more likely to connect samples from the same group. The test rejects the null if the number of between-group edges is significantly less than expected.

To be precise, we let **x**_1_,**x**_2_,…,**x**_*n*_ and **y**_1_,**y**_2_,…,**y**_*m*_ be i.i.d. samples from two multivariate distributions *F*_**X**_ and *F*_**Y**_, respectively. We first pooled samples from two groups and indexed them by 1,2,…,*N*=*n*+*m*. A MST is then constructed on the pooled samples using Kruskal’s algorithm [22]. Unless otherwise specified, *G* represents the MST (or other similarity graphs) as well as the set of all edges, and |*G*| denotes the total number of edges. To illustrate the technical details, we adopted the notations from Chen and Friedman’s paper. Let *g*_*i*_=0 if sample *i* is from group **X** and *g*_*i*_=1 otherwise. For the edge *e* connecting samples *i* and *j*, i.e., *e*=(*i*,*j*), we define:

5 $$ J_{e} = \left\{\begin{array}{ll} 0 &\text{if \(g_{i}\neq g_{j}\)}\\ 1 &\text{if \(g_{i}=g_{j}=0\)}\\ 2 &\text{if \(g_{i}=g_{j}=1\)}\\ \end{array},\right.  $$

and

6 $$ R_{k}=\sum\limits_{e\in G}I_{J_{e}=k}, k=0, 1, 2.  $$

Here *R*_1_ and *R*_2_ represent the numbers of edges connecting samples from same group, and *R*_0_ stands for number of edges connecting samples from different groups. The new test statistic simply quantifies the deviation of (*R*_1_,*R*_2_) from their expected values under true *H*_0_. It has the following quadratic form:

7 $$ S=(R_{1}-\mu_{1}, R_{2}-\mu_{2})\Sigma^{-1}\left(\begin{array}{l} R_{1}-\mu_{1} \\ R_{2}-\mu_{2} \end{array}\right),  $$

where *μ*_1_=*E*(*R*_1_), *μ*_2_=*E*(*R*_2_) and *Σ*=*V*((*R*_1_,*R*_2_)^*T*^) have the following expressions (see the Appendix of Chen and Friedman’s paper for detailed proof):

$$ {}\begin{aligned} \mu_{1} &=|G|\frac{n(n-1)}{N(N-1)}, \\ \mu_{2} &=|G|\frac{m(m-1)}{N(N-1)}, \\ \Sigma_{11} &=\mu_{1}(1\,-\,\mu_{1})+2C\frac{n(n-1)(n-2)}{N(N-1)(N-2)}+(|G|(|G|-1)\\&-2C)\frac{n(n-1)(n-2)(n-3)}{N(N-1)(N-2)(N-3)},\\ \Sigma_{22} &=\mu_{2}(1\,-\,\mu_{2})+\!2C\frac{m(m-1)(m-2)}{N(N-1)(N-2)}+(|G|(|G|-1)\\&-2C)\frac{m(m-1)(m-2)(m-3)}{N(N-1)(N-2)(N-3)}, \\ \Sigma_{12} &=(|G|(|G|\,-\,1)\,-\,2C)\frac{nm(n\,-\,1)(m\,-\,1)}{N(N\,-\,1)(N\,-\,2)(N\,-\,3)}-\!\mu_{1}\mu_{2}, \end{aligned}  $$

where $C=\frac {1}{2}{\sum \nolimits }_{i=1}^{N}|G_{i}|^{2}-|G|$, and *G*_*k*_ stands for the subgraph in *G* that includes all edges that connect to node *k*. It was proved that under the permutation null hypothesis, *S* asymptotically follows a Chi-square distribution with 2 degrees of freedom [9]. The *p*-value approximation generally works well under relatively small sample size, for instance, when min(*n*,*m*)=20. In their work, Chen and Friedman also suggested that the use of k-MST graphs (e.g., 3-MST or 5-MST) may lead to a better approximation of *p*-value in practice.

It is noteworthy to mention that Chen and Friedman’s method was developed for two-group comparison. In the case of multiple groups, a sequence of pairwise comparisons need to be conducted. Recently, we extended Chen and Friedman’s test to multiple-group case and proposed an overall test to compare more than two groups simultaneously. In our technical report [23], it was proved that the test statistics for *p* groups asymptotically follows a Chi-square distribution with p degrees of freedom under mild regularity conditions. To be precise, for an edge *e* in graph *G*, we let

$$ {}\begin{aligned} A_{e} & = \{e\} \cup \{e'\in G: e' \text{ and } e \text{ share a node}\}, \\ B_{e} & = A_{e}\! \cup\! \{e^{\prime\prime} \!\in G\! : \exists~e' \!\in A_{e}, \text{ such that } e^{\prime\prime} \text{ and } e' \text{ share a node}\}, \end{aligned}  $$

then the following theorem can be derived:

#### **Theorem 1**

If |*G*|=*O*(*N*), ${\sum \nolimits }_{k=1}^{N}|G_{k}|^{2}-\frac {4|G|^{2}}{N}=O(N)$, |*A*_*e*_||*B*_*e*_|=*o*(*N*^3/2^), ${\lim }_{N\rightarrow \infty }\frac {N_{i}}{N}=\lambda _{i}\in (0, 1)$, then

$${}S\!:=\!(\!R_{1}-\mu_{1}, R_{2}-\mu_{2}, \ldots, R_{p}-\mu_{p}\!)\Sigma^{-1}\!\!\begin{pmatrix} R_{1}\,-\,\mu_{1} \\ R_{2}\,-\,\mu_{2} \\ \cdots \\ R_{p}\,-\,\mu_{p}\!\end{pmatrix}\!\!\longrightarrow \chi^{2}_{p}, $$

where *i*=1,…,*p* is the group index.

The expected values and covariance matrix can be derived as in (7):

$$ {}\begin{aligned} \mu_{k, 1\leq k\leq p} &=|G|\frac{n_{k}(n_{k}-1)}{N(N-1)}, \\ \Sigma_{kk, 1\leq k\leq p} &=\mu_{k}(1-\mu_{k})+2C\frac{n_{k}(n_{k}-1)(n_{k}-2)}{N(N-1)(N-2)}+(|G|(|G|-1)\\&-2C)\frac{n_{k}(n_{k}-1)(n_{k}-2)(n_{k}-3)}{N(N-1)(N-2)(N-3)},\\ \Sigma_{jk, 1\leq j\neq k\leq p} &=(|G|(|G|-1)-2C)\frac{n_{j}n_{k}(n_{j}-1)(n_{k}-1)}{N(N-1)(N-2)(N-3)}-\mu_{j}\mu_{k}, \end{aligned}  $$

where $N={\sum \nolimits }_{k=1}^{p}n_{k}$ and $C=\frac {1}{2}{\sum \nolimits }_{i=1}^{N}|G_{i}|^{2}-|G|$. The detailed proof for Theorem 1 can be found in the Appendix of Zhang et al. (2017) [23].

#### Simulation study: edge-count test versus two existing approaches

We performed a simulation study to empirically compare the edge-count test with two existing methods based on Pearson’s correlation and mutual information. Particularly, we considered the following linear setting and nonlinear setting, where *X* and *Y* represent the expression levels of two genes and subscripts “1” and “2” stand for two conditions:

- Linear setting: $(X_{1}, Y_{1})^{T}\sim \mathrm {N}\left [\left (\begin {array}{l} 0\\ 0 \end {array}\right), \left (\begin {array}{ll} 1 & \rho \\ \rho & 1 \end {array}\right)\right ]$, $(X_{2}, Y_{2})^{T}\sim \mathrm {N}\left [\left (\begin {array}{l} 0\\ 0 \end {array}\right), \left (\begin {array}{cc} 1 & \rho +\Delta \\ \rho +\Delta & 1 \end {array}\right)\right ]$, where *ρ*=0.3, *Δ*∈{0.1,0.2,…,0.6}.
- Nonlinear setting: *X*_*i*_∼Uniform(−2,2), $Y_{i}=X_{i}^{2}+\epsilon _{i}$, $\epsilon _{i}\sim \mathrm {N}\left (0, \sigma _{i}^{2}\right)$, *i*=1,2, *σ*_1_=0.5, *σ*_2_=*σ*_1_+*Δ*, *Δ*∈{0.1,0.2,…,0.6}.

For each setting, we generated 1,000 data sets with sample sizes *n*_1_=*n*_2_=100 and three approaches were applied to test for the difference between two joint distributions. For edge-count test, we took 3-MST based on Euclidean distance and computed the *p*-value using Chi-square approximation. The R package *infotheo* [24] was used to estimate the entropies of *X*_*i*_ and *Y*_*i*_, as well as the mutual information between *X*_*i*_ and *Y*_*i*_, *i*=1,2. To evaluate the significance of the mutual information change, we performed a Fisher’s z transformation introduced in Zhang et al. (2012) [25]. To be precise, let *H*(*X*_*i*_) be the entropy of variable *X*_*i*_, and *I*(*X*_*i*_,*Y*_*i*_) be the mutual information between *X*_*i*_ and *Y*_*i*_, then the transformed *z*_*i*_ given below approaches to a standard normal distribution with variance $\frac {1}{n_{i}-3}$:

$$z_{i}=\frac{1}{2}\log\frac{1+I^{*}(X_{i},Y_{i})}{1-I^{*}(X_{i},Y_{i})},$$

where $I^{*}(X_{i},Y_{i})=\frac {I(X_{i},Y_{i})}{H(X_{i})+H(Y_{i})}$. The *p*-value can then be obtained by a two-sample z test, i.e.,

$$p\text{-value}_{MI}=2P\left(Z>\frac{|z_{1}-z_{2}|}{\sqrt{\frac{1}{n_{1}-3}+\frac{1}{n_{2}-3}}}\right).$$

**Fig. 3:**
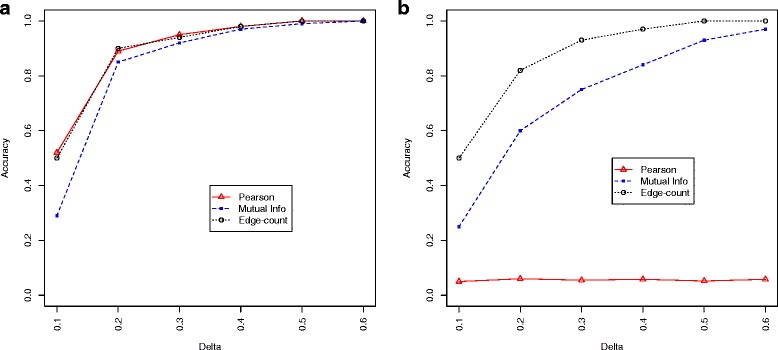
Performance comparison of three different methods under: (**a**) linear setting and (**b**) nonlinear setting. The x-axis is the value of *Δ*, and y-axis is the true positive rate based on 10,000 replications

For each data set, we conducted a quantile normalization to match the marginals and tested the hypothesis at *α*=0.05 ($H_{0}: \pmb {F}^{*}_{1}(X_{1},Y_{1})=\pmb {F}^{*}_{2}(X_{2},Y_{2})$) with three different methods, where $\pmb {F}^{*}_{i}$ represented the joint distribution of (*X*_*i*_,*Y*_*i*_) after the marginal matching. The accuracy (true positive rate) of each method under each setting was summarized in Fig. 3. As we can see, all the three methods achieved good accuracy in the linear setting (except in the subtle case of *Δ*=0.1). The Pearson’s correlation and edge-count test performed slightly better than the mutual information. For the nonlinear (quadratic) setting, the edge-count test substantially outperformed the other two methods, while the Pearson’s method completely failed to identify the difference.

Our simulation study demonstrated the capability of our edge-count test in capturing both linear and nonlinear changes. Generally, the edge-count test performs similarly well as Pearson’s correlation and mutual information under linear setting but achieves significantly better sensitivity for nonlinear setting.

## Results

In this section, we applied the two-step pipeline to search DCE genes in four subtypes of breast cancer using the Cancer Genome Atlas (TCGA) data. Four gene sets, including two KEGG gene pathways and two MSigDB hallmark gene sets, were used as illustrative examples. We validated our findings by the large-scale METABRIC breast cancer data.

### Data preparation

In TCGA, each subject is represented by multiple molecular data types including gene expression, genotype (SNP), exon expression, MicroRNA expression, copy number variation, DNA methylation, somatic mutation, and protein expression [3, 26]. We only used the gene expression (RNA-seq) data in this study. The TCGA transcriptome profiling data was downloaded through Genomic Data Commons (GDC) portal in January 2017. The expression level of each gene was quantified by the count of reads mapped to the gene. The quantifications were done by software *HTSeq* of version 0.9.1 [27] and the count data were log-transformed for further processing. We excluded 43 subjects from the analysis including 12 male subjects and 31 subjects with more than 1% missing values. In addition, we removed the effects due to different age groups and batches using a median-matching and variance-matching strategy [28]. For example, the batch effect can be removed in the following way:

$$g^{*}_{ijk}=M_{i}+(g_{ijk}-M_{ij})\frac{\hat{\sigma}_{g_{i}}}{\hat{\sigma}_{g_{ij}}},$$

where *g*_*ijk*_ refers to the expression value for gene *i* from sample *k* in batch *j* (*j*=1,2,…,*J*;*k*=1,2,…,*n*_*j*_), *M*_*ij*_ represents the median of $g_{ij}=(g_{ij1},\ldots,g_{ijn_{j}})\phantom {\dot {i}\!}$, *M*_*i*_ refers to the median of *g*_*i*_=(*g*_*i*1_,…,*g*_*iJ*_), $\hat {\sigma }_{g_{i}}$ and $\hat {\sigma }_{g_{ij}}$ stand for the standard deviations of *g*_*i*_ and *g*_*ij*_, respectively.

The remaining 959 samples were further classified into five subtypes according to two molecular signatures, namely *PAM50* [29] and *SCMOD2* [30]. The two classifications were implemented separately using R package *genefu* [31] and we obtained 530 subjects with concordant classification by two classifiers. The resulting set contains 221 subjects in luminal A group, 119 in luminal B group, 74 in her2-enriched group, 105 in basal-like group and 11 in normal-like group. The normal-like group was excluded from the analysis due to the low sample size and only four subtype groups were considered.

Finally, we perform a quantile normalization [32] for each group separately, so that the marginal distributions of all the genes match across groups. The purpose of quantile normalization is to avoid the rejection of *H*_0_ due to marginal difference (differential expression) instead of different dependency patterns (differential co-expression).

### Some illustrative examples

We illustrated the new method using four molecular pathways, including the cell cycle and ERBB pathways from KEGG database, as well as the JAK-STAT and TGF-beta signaling pathways from MSigDB database. All the selected pathways play critical roles in the initiation and progression of many human cancers. For instance, KEGG cell cycle pathway contains 128 genes that co-regulate cell proliferation, including *ATM*, *RB1*, *CCNE1* and *MYC*. Abnormal regulation among these genes may cause the over proliferation of cells and an accumulation of tumor cell numbers. The ERBB pathway in KEGG database consisted of 87 genes including important proto-oncogenes and tumor suppressors such as *PIK3C*, *KRAS* and *STAT5*. It is known that ERBB pathway is closely related to the development of a wide variety of types of tumor. Especially, the excessive signaling of growth factor receptors *ERBB1* and *ERBB2* are critical factors in the malignancy of solid tumor [3]. The JAK-STAT signaling pathway and TGF-beta signaling pathway were also known to play critical roles in tumor suppression and cancer metastasis. For instance, TGF-beta can modulate processes such as cell invasion, immune regulation, and microenvironment modification that cancer cells may exploit to their advantage [33].

For each subtype group, we first computed the distance correlation matrix and corresponding *p*-value matrix for all gene pairs (see Methods section for details). A Benjamini-Hochberg (BH, [34]) procedure with FDR≤0.05 was then applied to screen out uncorrelated genes. A gene pair was deemed as uncorrelated if the adjusted *p*-values in four subtypes are all above 0.05. This screening resulted in a total of 487 correlated gene pairs in cell cycle pathway, 359 in ERBB pathway, 592 in JAK-STAT signaling pathway and 440 in TGF-beta signaling pathway. These four reduced sets of gene pairs were used as the search space for the testing step.

**Fig. 4:**
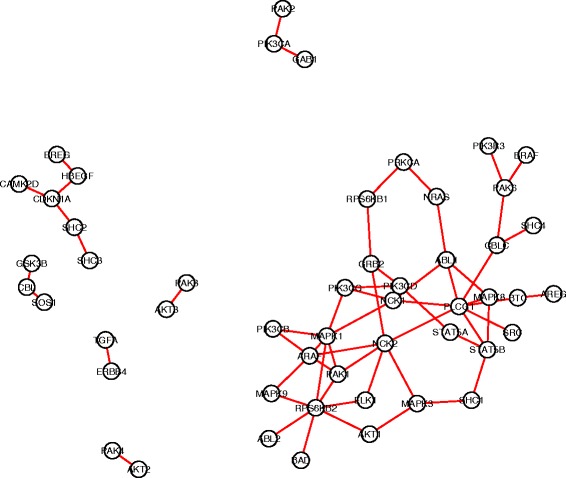
The collection of differentially co-expressed gene pairs in KEGG cell cycle pathway. A connection between two genes represents significant DCE by the edge-count test

**Fig. 5:**
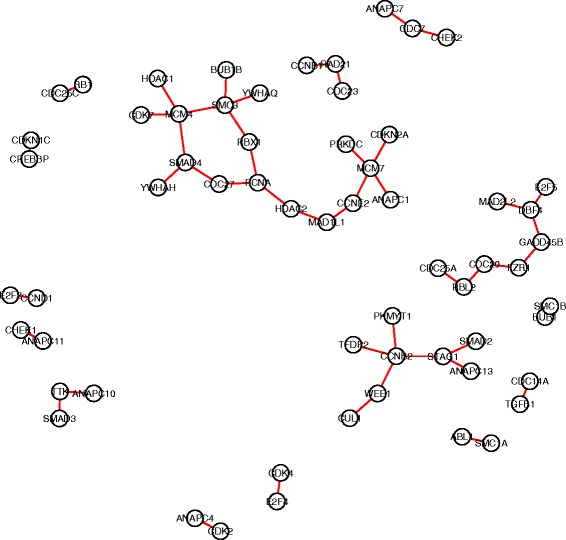
The collection of differentially co-expressed gene pairs in KEGG ERBB pathway. A connection between two genes represents significant DCE by the edge-count test

**Fig. 6:**
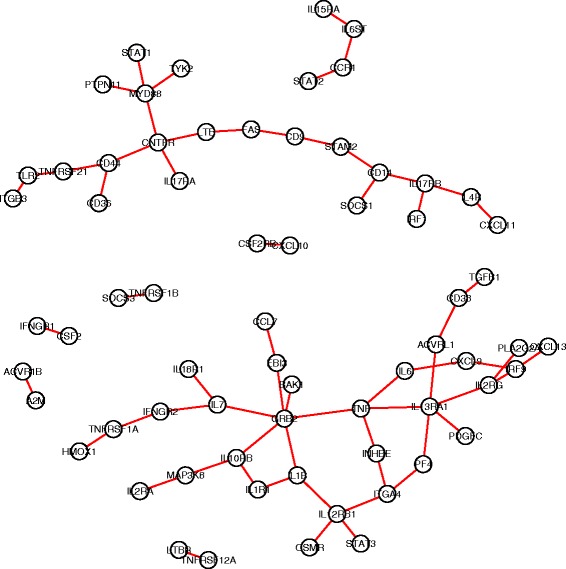
The collection of differentially co-expressed gene pairs in MSigDB JAK-STAT pathway. A connection between two genes represents significant DCE by the edge-count test

**Fig. 7:**
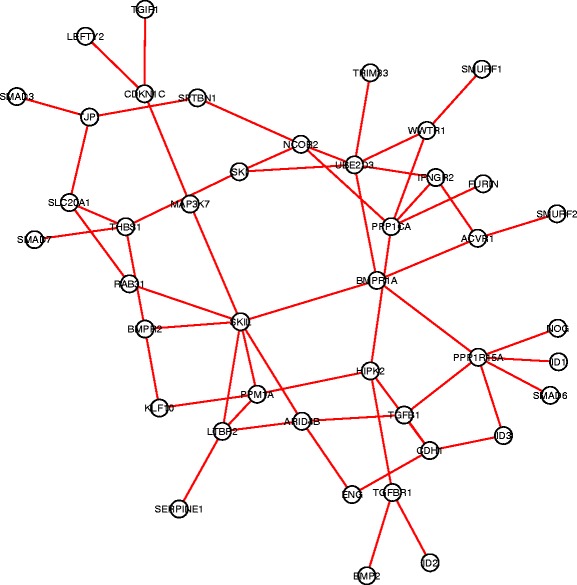
The collection of differentially co-expressed gene pairs in MSigDB TGF-beta pathway. A connection between two genes represents significant DCE by the edge-count test

For each gene pair in the search space, we carried out hypothesis tests to compare the co-expression patterns in each pair of subtypes (totally $\binom {4}{2}=6$ comparisons). An edge-count test with 3-MST was implemented, followed by a BH procedure with FDR≤0.05 for multiplicity adjustment. Finally, we identified 120 DCE gene pairs in cell cycle pathway, 94 in ERBB pathway, 122 in JAK-STAT signaling pathway and 102 in TGF-beta signaling pathway. Figures 4, 5, 6, 7 showed the four DCE networks, where each edge indicated a DCE gene pair in four subtypes. It should be noted that the networks we presented here are different from the regular gene co-expression networks, instead, each network represents a collection of gene pairs that are differentially co-expressed under different conditions. When interpreting the clusters in the networks, one reasonable hypothesis could be that they represent groups of genes that are significantly co-expressed in some condition/conditions but not in others. For instance, we found that genes *MYD88, STAT1, TYK2, PTPN11, CNTFR, IL17RA, LTE* and *CD44* (highly connected in Fig. 6) exhibited a much stronger co-expression in the basal-like subtype than the other three subtypes, according to the distance correlation matrices in four subtypes. In practice, one may use our pipeline to infer the differentially co-expressed network, and then focus on a subnetwork (subset of genes) of interest by investigating the co-expressions in different conditions, either numerically or graphically.

**Fig. 8:**
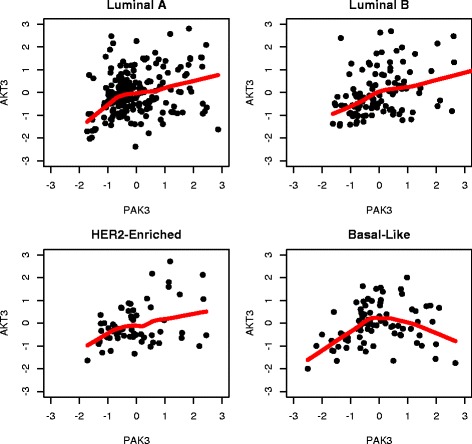
An example of identified DCE genes: the co-expression graphs between *PAK3* and *AKT3* (KEGG cell cycle pathway) in four breast cancer subtypes

**Fig. 9:**
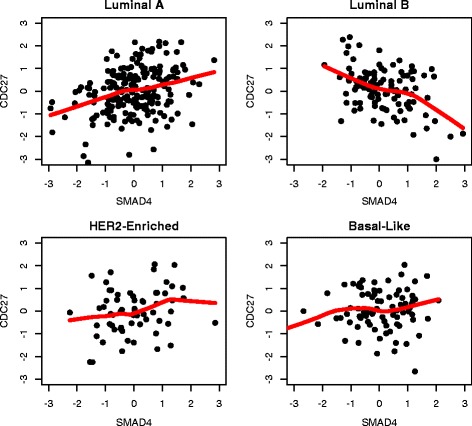
An example of identified DCE genes: the co-expression graphs between *SMAD4* and *CDC27* (KEGG ERBB pathway) in four breast cancer subtypes

Two examples of the identified DCE gene pairs were shown in Figs. 8 and 9. Figure 8 suggested that the co-expression of genes *PAK3* and *AKT3* in basal-like group was substantially different from those in the other groups. In Fig. 9, genes *SMAD4* and *CDC27* exhibited a negative co-expression in the luminal B group, which was not observed in luminal A, her2-enriched or basal-like group.

### Comparison with Pearson’s correlation method

**Fig. 10:**
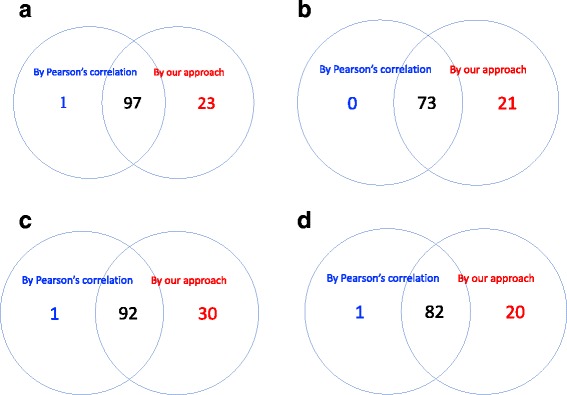
The Venn diagram showing the agreement between Pearson’s correlation method and the proposed method for each pathway: (**a**) KEGG cell cycle pathway; (**b**) KEGG ERBB pathway; (**c**) MSigDB JAK-STAT signaling pathway and (**d**) MSigDB TGF-beta signaling pathway

**Fig. 11:**
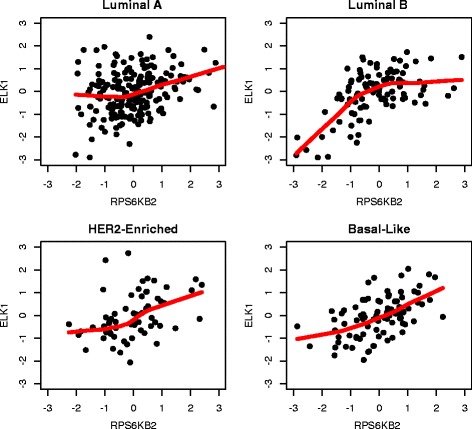
An example of DCE gene pair identified by new approach but missed by Pearson’s correlation method: the co-expression graphs between *RPS6KB2* and *ELK1* (cell cycle pathway) in four subtypes

**Fig. 12:**
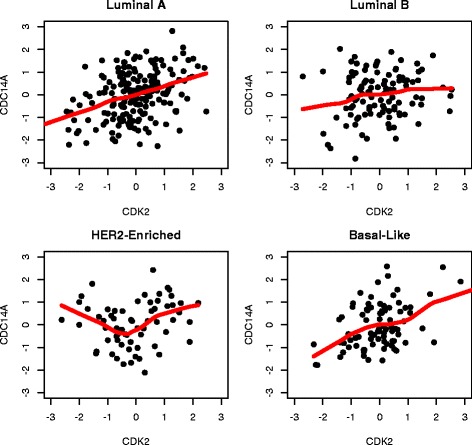
An example of DCE gene pair identified by new approach but missed by Pearson’s correlation method: the co-expression graphs between *CDK2* and *CDC14A* (ERBB pathway) in four subtypes

To benchmark our new method, we compared it with the DCE search based on Pearson’s correlation, as introduced in the Background section. A two-sample z-test with Fisher’s z-transformation was conducted, followed by a BH procedure with FDR≤0.05 for fair comparison. By Pearson’s correlation method, a total of 98 DCE gene pairs were identified in cell cycle pathway, 73 in ERBB pathway, 93 in JAK-STAT signaling pathway and 83 in TGF-beta signaling pathway. The agreement between the two approaches was summarized using Venn diagrams in Fig. 10. It can be seen that almost all the DCE genes by Pearson’s method were also captured by the new approach, but a significant number of gene pairs captured by the new approach were missed by the Pearson’s correlation method. Two gene pairs of such were provided as examples in Figs. 11 and 12. The different association patterns between genes *RPS6KB2* and *ELK1* in four groups were shown in Fig. 11, where it could be seen that in luminal B subtype, the two genes were positively associated when *RPS6KB2* was underexpressed, but the expression of *ELK1* became stabilized when *RPS6KB2* was overexpressed. By Pearson’s correlation method, however, none of the *p*-values was significant. There were two *p*-values (after adjustment) that were highly significant by the edge-count test: *p*=9.4×10^−4^ for $H_{0}: \pmb {F}^{*}_{luminal~A}=\pmb {F}^{*}_{luminal~B}$ and *p*=1.1×10^−3^ for $H_{0}: \pmb {F}^{*}_{basal-like}=\pmb {F}^{*}_{luminal~B}$. Likewise, as shown in Fig. 12, genes *CDK2* and *CDC14A* exhibited a V-shape co-expression in her2-enriched group, but not in the other groups. These examples indicated that our new method dominates the prevailing Pearson’s correlation method in searching DCE genes, therefore may reveal additional clues for understanding the changes in gene regulation mechanisms of different phenotypes.

### Validation by METABRIC data

**Fig. 13:**
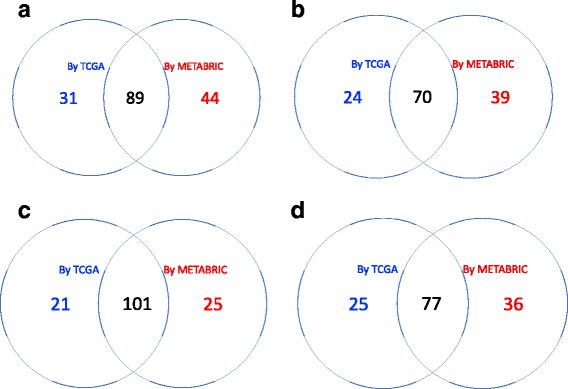
The Venn diagram showing the agreement between TCGA data and METABRIC for each pathway: (**a**) KEGG cell cycle pathway; (**b**) KEGG ERBB pathway; (**c**) MSigDB JAK-STAT signaling pathway and (**d**) MSigDB TGF-beta signaling pathway

**Fig. 14:**
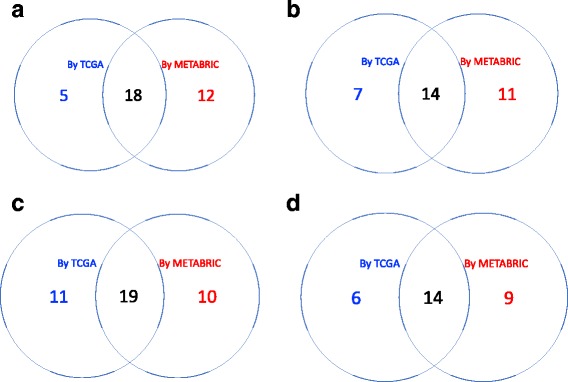
The Venn diagram showing the agreement between two sets of nonlinear DCE gene pairs (identified by edge-count test but missed by Pearson’s method) by TCGA data and METABRIC data: (**a**) KEGG cell cycle pathway; (**b**) KEGG ERBB pathway; (**c**) MSigDB JAK-STAT signaling pathway and (**d**) MSigDB TGF-beta signaling pathway

To validate the identified sets of DCE gene pairs, we repeated the two-step procedure to large-scale data cohort, namely the METABRIC data [35]. The METABRIC data set contained molecular profiles for 2506 breast cancer samples and each sample has been assigned a subtype based on PAM50 signature. In our analysis, we included 700 samples in luminal A group, 475 in luminal B group, 224 in her2-enriched group and 209 in basal-like group. After the distance correlation screening and quantile normalization for each gene, we applied two methods, namely the Pearson’s correlation and edge-count test, to search DCE gene pairs in the four aforementioned gene sets. Same thresholds of FDR cutoff for distance correlation screening and edge-count test were used as in the TCGA analysis. With the METABRIC data, we identified four sets of DCE gene pairs for four pathways and Fig. 13 summarized the comparison between TCGA data and METABRIC data. The agreement between the two data sets ranged from 64.2 to 80.2% for four pathways, indicating a satisfactory reproducibility of our method. In addition, we compared two DCE sets of the nonlinear type that were identified by edge-count test but missed by Pearson’s method. As can be seen from Fig. 14, these two data sets also achieved a good agreement on the nonlinear DCE pairs. For instance, out of 23 nonlinear DCE pairs using TCGA data, 18 were confirmed by the METABRIC data.

## Discussion

In this article, we developed a nonparametric method to effectively identify variability in gene co-expression pattern among multiple phenotypes. Our work presents novelty in two aspects. Firstly, we dropped the assumption of joint normality between genes and directly test if a gene pair follow the same joint distribution over different phenotypes. By a graph-based approach, the comparison between multivariate distributions was transformed to an edge-count test which is easy to implement. The statistical test used in this study is fully nonparametric and it rejects null hypothesis under different types of differential co-expressions including linear and nonlinear types. By a real life application, we demonstrated how the proposed test is better able to capture the DCE genes as compared to the Pearson’s correlation method.

Second, to make the test applicable to large-scale data, we employed a distance correlation measure to filter out all the noncoexpressed gene pairs prior to the testing step. One shortcoming of the edge-count test is that it requires the calculation of a similarity graph that connects all the samples. For example, in our analysis of the breast cancer data, a 3-MST (union of three non-overlapping MSTs) was used as the similarity graph. Under large number of genes, this step can be computationally expensive. As a well accepted fact in biology, most gene co-expression networks are overall sparse, although they might be locally dense, hence the co-expression screening step should considerately reduce the search space. In the example of KEGG cell cycle pathway, the search space was reduced from more than 8000 gene pairs to less than 500.

Throughout this paper, we have focused on the study of co-expression between two genes. Nevertheless, it is noteworthy that the proposed test can be readily applied to multiple-gene cases. In fact, Chen and Friedman’s test, as well as the multi-group extension, is merely built upon a similarity graph connecting all the samples, and the construction of graph depends only on the interpoint distances regardless of the dimension [9]. In practice, one can simply use Euclidean norm as the interpoint distance and construct the similarity graph such as MST or k-MST. Additionally, because of the flexibility of our approach, one can also explicitly test for the difference in a higher-order interaction such as three-way gene co-expression, by properly controlling all the marginals and lower-order interactions.

## Conclusions

Differential co-expression analysis is critical for the identification of disease-related factors. Motivated by the fact that nonlinear co-expressions generally exist in cellular regulations, we develop a new nonparametric method for DCE analysis, which measures and compares gene co-expressions in linear and nonlinear aspects. Our method does not rely on any assumption regarding the probability distributions of the genes being studied, but it generally tests the equality of two or multiple co-expression patterns through a powerful graph-based test. For practical consideration, we suggest a screening step based on distance correlation to tackle the computational burden for large-scale data. The proposed computational procedure can also be applied to other similar bioinformatics problems such as the differential co-methylation analysis [36, 37] and differential gene set analysis [38, 39].

## Abbreviations

BH: Benjamini-Hochberg
DC: Distance correlation
DCE: Differential co-expression or differentially co-expressed
DE: Differential expression or differentially expressed
MST: Minimum spanning tree
TCGA: The cancer genome atlas
